# T2-fluid attenuated inversion recovery fat-suppressed mismatch in the identification and characterization of lesions related to radiologically isolated syndrome

**DOI:** 10.1093/bjrcr/uaae028

**Published:** 2024-08-13

**Authors:** Darin T Okuda, Christine Lebrun-Frenay

**Affiliations:** Department of Neurology, Neuroinnovation Program, Multiple Sclerosis & Neuroimmunology Imaging Program, The University of Texas Southwestern Medical Center, Dallas, TX, 75390-8806, United States; The University of Texas Southwestern Medical Center, Peter O’Donnell Jr. Brain Institute, Dallas, TX, 75390, United States; Neurology, Université Nice Cote d’Azur, UR2CA-URRIS CRCSEP CHU Nice Pasteur, Nice, 06002, France

**Keywords:** radiologically isolated syndrome, MRI, T2-FLAIR mismatch, fat suppressed, central vessel sign

## Abstract

The radiologically isolated syndrome is defined by the presence of incidentally identified T2-weighted hyperintense lesions, highly suggestive of central nervous system demyelination, following an MRI study that is performed for reasons other than for the investigation of symptoms related to multiple sclerosis (MS). These individuals also have no evidence of prior neurological symptoms associated with inflammatory demyelination and no alternative explanation for the observed MRI findings. Recently, the introduction of novel imaging techniques such as the “central vein sign” has improved lesion specificity for MS. In addition, the observation of T2-fluid attenuated inversion recovery (FLAIR) mismatch characteristics associated with gliomas and in those with MS with a higher disease burden appear to provide morphological data that relate to disease severity. The value of T2-FLAIR mismatch characteristics in discrete multi-focal lesions has not yet been well defined. Here, we present the value of a fat-suppressed T2-FLAIR sequence in the identification and characterization of T2-weighted hyperintensities resulting from inflammatory demyelination.

## Introduction

The radiologically isolated syndrome (RIS) currently represents the earliest detectable pre-clinical phase of multiple sclerosis (MS) in select individuals.^1^ Incidental T2-weighted hyperintense lesions, highly suggestive of an MS origin, may be identified in healthy individuals when an MRI study is performed for reasons other than the investigation of central nervous system (CNS) inflammatory demyelination (eg, headache, trauma, etc.). Younger age, the presence of cerebrospinal fluid (CSF)-restricted oligoclonal bands, infratentorial/spinal cord involvement, and temporal changes on MRI are risk factors for the development of a first demyelinating event in subjects.^2^

Neurologists routinely consult on individuals with abnormal MRI studies of the brain following the report of clinical symptoms. Whether abnormal features are observed in this setting or those with no definable symptoms related to MS, there is a risk for the misclassification of lesions observed, resulting in an inaccurate diagnosis. Novel imaging metrics have been pursued to enhance the specificity of the lesions observed on MRI with the “central vein sign,” the observation of a venule located centrally within a lesion and observed in at least 2 planes of view on 1.5 or 3T platforms, improving upon the classification of lesions. This finding is also validated in paediatric and adult populations.^3^ However, uniform access to the required iron-sensitive susceptibility-based sequences and post-processing expertise may be limited within healthcare practices.

T2-fluid attenuated inversion recovery (FLAIR) mismatch has been proposed as an imaging metric for isocitrate dehydrogenase-mutant astrocytomas lacking 1p/19q codeletion with high specificity.^4^ Imaging features associated with this descriptor initially included the “presence or absence of complete/near-complete hyperintense signal on T2-[weighted imaging], and relatively hypointense signal on FLAIR except for a hyperintense peripheral rim.”^4^ Such findings also appear present in tumefactive MS,^5^ suggesting that other morphological differences within lesions may exist beyond currently applied techniques for clinical care.

Here, we describe a young man with a significant history of incidental anomalies within the brain that were identified after an MRI study was performed for a concussion sustained during a collegiate football game. The potential value of T2-FLAIR mismatch through the utilization of a T2-FLAIR fat-suppressed sequence that may provide greater morphological information in the identification and characterization of asymptomatic focal lesions associated with CNS autoimmune demyelination is presented.

## Consent

Written informed consent was obtained from the person described in this report for publication, including use of the accompanying images.

## Clinical presentation

A 20-year-old right-handed Black man with no significant past medical history was evaluated for headache after sustaining a concussion without loss of consciousness during a college football game. Chronic headache in the absence of nausea and vomiting was described with associated photo- and phonophobia. Modest treatment benefit with over-the-counter acetaminophen and ibuprofen was described. He denied prior symptoms of vision loss, weakness involving the extremities, sensory abnormalities, or difficulties with bulbar function. In addition, there was no history of prior mood alterations, bowel or bladder dysfunction, or gastrointestinal abnormalities.

**Figure 1.**
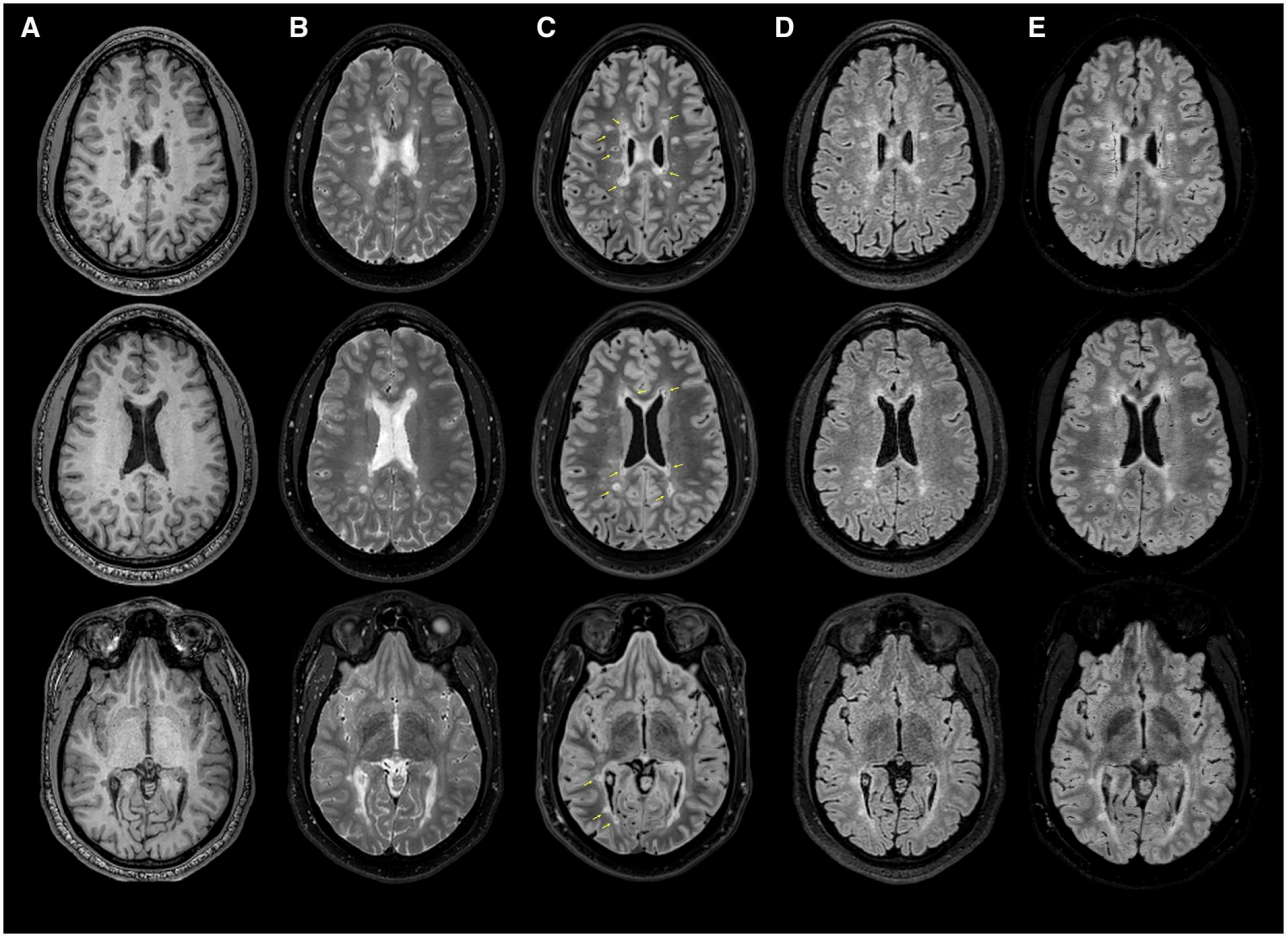
Select sequences from a 3-Tesla MRI of the brain. (A) Axial T1-weighted and (B) T2-weighted images of the brain revealing multi-focal regions of signal abnormality in locations typical for multiple sclerosis (MS). (C) Axial T2-fluid attenuated inversion recovery (FLAIR) fat-suppressed images demonstrating regions of T1-hypointensity within lesions (yellow arrows). Note the different characteristics in lesions from the FLAIR imaging sequence (D) and the fat-suppressed T2-FLAIR mismatch (B compared to C). (E) Axial FLAIR star MRI images of the brain highlighting the “central vein sign” within selected lesions in (C). The number of lesions with central vessels present suggest an origin of MS.

On neurological examination, vital signs were within normal limits. His general appearance revealed a healthy, athletic-looking individual who was in no acute distress. Deficiencies in cranial nerve, motor, sensory (primary modalities and cortical sense), coordination, and gait were not observed. A complete blood count and comprehensive metabolic panel were unrevealing. Antinuclear antibody testing was normal. The presence of >2 CSF-restricted oligoclonal bands was also observed. Brain MRI demonstrated non-gadolinium-enhancing multi-focal regions of high-signal abnormality throughout the supratentorial region with an appearance highly suggestive of inflammatory demyelination. Imaging findings from a fat-suppressed FLAIR sequence revealed the presence of hypointense regions within some of the observed T2-weighted hyperintensities, but not all of them. FLAIR star (FLAIR*) imaging revealed the presence of central vessels within focal areas of high signal abnormality (Figure 1).

## Discussion

The reported clinical experience was significant for identifying incidental anomalies within the brain that appeared highly typical for features related to inflammatory demyelination. The imaging findings were recognized following a traumatic event on the football field with no historical or recent clinical correlation despite the observation of an intramedullary lesion within the cervical spinal cord.

Given the high sensitivity of MRI, an associated risk exists for the misclassification of subjects, particularly when T2-weighted lesion morphologies appear to be related to mixed origins. The value of the central vessel sign has improved the specificity for T2-weighted hyperintensities related to CNS demyelination in those with RIS, both in the diagnosis and on follow-up imaging studies to confirm that newly developed T2-weighted lesions are related to inflammatory autoimmune injury.^6^ Considerations for other potential causes of the observed lesions included non-specific white matter disease related to chronic migraine or microvascular sequelae, vasculitis, etc. The observation of more than 6 lesions containing a central vessel within the FLAIR* sequence, in the absence of a better explanation for the MRI findings, suggested a CNS autoimmune inflammatory cause. Interestingly, many but not all T2-weighted hyperintense foci contained T2-FLAIR mismatch characteristics, and the greater appreciation of such characteristics may likely be the result of the fat-suppressed sequence applied, in comparison to the standard FLAIR sequence (Figure 1). This observation suggests heterogeneity in the morphology of lesions as components within the lesion appear to possess a similar longitudinal relaxation curve to that of CSF. The exact reasons for this observation are unclear, given the lack of histopathological correlates, and the findings when compared to T1-weighted imaging data appear to offer additional insights into the morphology of lesions. However, this mismatch, without fat suppression, has been observed in those with MS^5^ with a higher disease burden but has not yet been well defined in discrete multi-focal lesions.

Overall, identifying this radiological characteristic involving the use of a fat-suppressed T2-FLAIR sequence may be beneficial in improving the specificity of lesions related to MS and the presence may provide insights into the magnitude of injury within lesions when distinct spatial characteristics associated with inflammatory CNS demyelination are seen. However, it is important to highlight that the degree of specificity in typical MS lesions is unknown as such imaging features may also be present in other conditions. In addition, we speculate that mismatched lesions may also be associated with distinct changes in 3-dimensional shape and volume that could forecast persistent T1 “black holes,” indicating a higher degree of axonal compromise.^7^ Certain race/ethnic groups may also be selectively vulnerable to such lesions.^8^ The images yielded from the T2-FLAIR fat-suppressed sequence do not require post-processing, gadolinium to enhance radiological characteristics, or technical skill and can be acquired in 5 minutes.

Recently, 2 randomized clinical trials for people with RIS in the United States (ARISE) along with Europe and Türkiye (TERIS) demonstrated the benefit of early treatment in preventing/delaying the onset of a first clinical event associated with CNS inflammatory demyelination.^9^^,^^10^ These studies showed the benefit of 2 approved disease-modifying therapies, dimethyl fumarate (Tecfidera^®^) and teriflunomide (Aubagio^®^), each having a distinct mechanism of action. Given that the original brand name products studied in the pivotal RIS therapeutic trials are now generic, along with the observed burden of disease within the brain and spinal cord, the subject within this report was treated with an anti-CD20 agent.

The expansion of new MRI techniques has improved the specificity of T2-weighted hyperintense lesions and the longitudinal evaluation of chronic active lesions via MRI. However, current limitations exist in the generalized access to these techniques along with expertise and the required support for image processing. The observations presented here create an opportunity for further research in exploring the value of T2-FLAIR fat-suppressed mismatched lesions not only in the context of RIS but also in MS along with other disease states in which non-specific white matter lesions are prominent.

## Learning points

- Incidental anomalies highly suggestive of multiple sclerosis may be present in individuals with no history of symptoms related to CNS demyelination.
- T2-FLAIR mismatch has been described in gliomas, tumefactive demyelination, and in those with a high MS disease burden, but characteristics within typical multi-focal lesions related to MS are unknown.
- A fat-suppressed T2-FLAIR sequence may be of value in the identification of T2-weighted hyperintensities related to inflammatory demyelination and may provide additional morphological details related to the degree of myelin and axonal damage.
- Data from a fat-suppressed T2-FLAIR sequence do not require post-processing techniques.
- The value of T2-FLAIR fat-suppressed findings in the characterization of other disease states in which white matter lesions are present is needed.
